# Measuring spatial accessibility and supply-demand deviation of urban green space: A mobile phone signaling data perspective

**DOI:** 10.3389/fpubh.2022.1029551

**Published:** 2022-10-21

**Authors:** Jingyuan Chen, Cheng Wang, Yunbin Zhang, Dan Li

**Affiliations:** Department of Urban and Rural Planning, School of Forestry and Landscape Architecture, Anhui Agricultural University, Hefei, China

**Keywords:** urban green space, mobile phone signaling data, spatial accessibility, supply-demand, Ga2SFCA method

## Abstract

The reasonable distribution of urban green space (UGS) is a topic that urban researchers have been exploring for a long time. Solving the imbalance between the supply and demand of UGS plays an important role in improving the health level of a city. This study examines the central urban area of Hefei as an example. We developed a modified Gaussian two-step floating catchment area method and used the path planning model of Gaode Map to evaluate the accessibility of UGS under different transportation modes and different time thresholds while integrating mobile phone signaling data. Additionally, a fine-scale analysis of the actual supply and demand relationship of UGS was conducted by integrating the accessibility evaluation results with the recreational situation of UGS to analyze the deviation of supply and demand to further discuss the spatial distribution equilibrium of UGS. The main conclusions are as follows. (1) The spatial distribution of UGSs in the central urban area of Hefei is uneven. Different time thresholds and different transportation modes have a significant impact on the UGS accessibility evaluation results. (2) With the increase in the time threshold or travel distance, the number of grids above the moderate accessibility level generally increases. The spatial distribution of the grids with moderate, high and highest accessibility level present different patterns of contiguous, clusters, and spots distribution. (3) After combining these results with the actual recreational situation of UGS, we found that the overall demand in the central urban area exceeds the supply at the 15-min threshold, while the overall supply exceeds the demand at the 30-min threshold. The grids with balanced supply and demand or more supply than demand have comprehensive parks with a moderate population density and strong road connectivity in the neighborhood. This study strengthens the data granularity and improves the accuracy of accessibility evaluation by integrating mobile phone signaling data with the path planning model of Gaode Map. Also, we evaluate the accessibility with multi-transport modes and different time thresholds, which can bring more practical guidance for optimizing the distribution of UGS.

## Introduction

With the acceleration of urbanization in China, residents' demand for urban green space (UGS) is growing (1). Meanwhile, as an important component of urban ecosystems (2), UGS can reduce the negative impact of environmental factors such as space pollution (3), noise (4), and high temperatures (5). Prolonged exposure to UGS helps promote the physical and mental health of residents (6) and reduces stress as well as the risk of obesity, stroke, and other diseases (7). Due to that the traditional evaluation of UGS is based on the traditional indicators of urban park area per capita, the greening rate and green coverage rate, it can be difficult to reflect the spatial distribution, quality, and fairness of the service function of UGS (8). In the context of limited urban construction land, it is of great theoretical and practical significance to evaluate the spatial distribution quality of the existing UGS.

Accessibility is an important index to measure the fairness of UGS spatial distribution. Accessibility refers to the potential of overcoming resistance, such as time and distance, to reach a certain place (9). In recent years, an increasing number of studies have introduced the concept of accessibility into the evaluation of UGS. Researchers often measure the accessibility of UGS by setting statistical indicators (10) and using the cost-weighted distance method (11) or the gravity model method (12). The statistical index method evaluates accessibility by counting the number of places and area of UGS in the region, or by counting the number of places and area of UGS within a certain buffer zone from the living area (13). This method is also known as the cumulative opportunities method (14) or the covering method (15). The disadvantage is that it does not take into account the resistance that residents encounter on their way to UGS, and it is difficult to reflect the accessibility within the UGS' actual service area. Based on remote sensing data, the cost-weighted distance method assigns different crossing resistances to different urban landscape types, and the accessibility is evaluated by calculating the cumulative resistance encountered by residents on their way to the UGS (8). The advantage is that this method takes into account the impact of resistance on accessibility, but the disadvantage is that it does not consider the attractiveness of UGS of different qualities and levels; hence, the accessibility calculation results cannot accurately reflect the actual recreational situation (16). In the calculation of accessibility, the gravity model (17) adopts the distance decay function and considers the service capacity and potential of UGS, so that the results can better reflect the impact of UGS' attraction on accessibility. However, the relationship between residents' demand and accessibility remains to be further explored (18). The two-step floating catchment area (2SFCA) method was first proposed by Radke in 2000 (19) and was further improved by Luo and Qi (20) in 2009. Accessibility decreases with the increase of the resistance encountered by the residents on their way to the UGS and increases with the increase of the area and quality of the UGS. Based on the 2SFCA, the accessibility of UGS can be calculated comprehensively and simply from the perspective of the interaction between the park supply and residential demand, which makes up for the shortcomings of the gravity model (21). Due to its advantages, this method has been widely used in accessibility evaluation (21–24), and many studies have tried to improve this method to promote the accuracy of the evaluation results (Table 1).

**Table 1:** Main extensions of the Gaussian two-step floating catchment area (2SFCA) method.

**Type**	**Form**	**Content**	**Document**
Improvement based on supply and demand factors	Three-step floating catchment area (3SFCA) method	The choice weight between the demand point and supply point is used to represent the competition effect between facilities.	Wan et al. (25)
	Modified 2SFCA method	The primary attenuation function is reused considering the suboptimal space configuration of facilities.	Delamater (26)
	Huff 2SFCA method	The Huff model is used to quantify the probability of people choosing facilities when considering the influence of facility supply factors on facility selection behavior.	Luo (27)
Improvement based on the search distance	Dynamic 2SFCA method	Different search distances are set by considering different demand points for facility supply.	Mcgrail and Humphreys (33)
	Variable 2SFCA method	The search radius of variable supply and demand elements is set to balance the supply and demand within the search radius.	Luo and Whippo (34)
	Multi-catchment sizes 2SFCA method	The search radius is set according to the size and quality of the supply factors.	Tong et al. (22)
Improvement based on the distance attenuation function	Enhanced 2SFCA method	The distance is segmented within the search radius.	Luo and Qi (20)
	Gravity 2SFCA method	The distance attenuation function of the gravity model is added within the search radius, such as the power function, exponential function, and logarithmic function.	Wang and Tang (29)
	Gaussian 2SFCA method	The Gaussian function-type distance attenuation function is added within the search radius.	Dai andWang (30)
	Kernel density 2SFCA method	The kernel density function-type distance attenuation function is added within the search radius.	Dai (31)
Improvement based on the travel mode	Multi-mode 2SFCA method	The weighted average time of multiple transportation modes is used to replace the time of the single transportation mode.	Yang et al. (23)

Some studies revealed the competition effect between supply facilities and proposed the 3SFCA model to address the fact that 2SFCA cannot reflect the actual supply and demand situation. Based on the traditional 2SFCA model, 3SFCA calculates the option value between each pair of demand points and supply facility points and measures the competitive effect of multiple supply points within the same demand point search radius (25). However, Delamater showed that 3SFCA overestimates the competitive effect among supply points while ignoring the availability of the facility resources (26). Therefore, a simpler Modified 2SFCA model was proposed, which introduced the attenuation function in the selection weight to reflect whether facility resources are fully utilized, simplifying the quantification process of competition effect between supply facilities. Luo et al. (27) introduced the Huff model to reflect the selection weights, and on this basis, proposed the Huff 2SFCA model, which quantified the probability of people choosing supply facilities with continuous weight functions, further improving the applicability of the 2SFCA model. In the original form of 2SFCA, distance decay is treated dichotomically (28): the accessibility within the search radius is the same, and the accessibility outside the search radius is 0. This method ignores the influence of the spatial distance on accessibility. Some studies used different distance decay functions to solve this problem. For example, Luo and Qi (20) proposed the optimized 2SFCA to segment the distance within the search radius. However, the segmentation of distance is greatly influenced by subjectivity to a certain extent, and the segmentation weight given to the distance will lead to discontinuous results at the segmentation boundary. Therefore, 2SFCA applies continuous functions such as the gravity model (29), kernel density function (30), and Gaussian function (31) to solve the above problems. The Gaussian function is an s-shaped attenuation, and the attenuation rate of accessibility with distance is slower in the near and far stages than in the middle, which is more consistent with the actual situation than the 2SFCA method. Therefore, it is widely used in the optimization of accessibility evaluation methods (32). The 2SFCA model also fails to consider multiple traffic modes comprehensively. Yang et al. (23) proposed the multi-mode 2SFCA model to solve this problem. This model uses the weighted average time of multiple transportation modes to replace the time of a single transportation mode, which can better reflect the heterogeneity of accessibility under different transportation modes. Taking the central urban area of Hefei as an example, this study optimized the 2SFCA model for the purpose of comparing the accessibility differences of UGS under various transportation modes. Mobile phone signaling data were used in the study and combined with the Gaode map path planning model to improve the shortcomings of poor timeliness and low accuracy of the evaluation results caused by the use of statistical data for evaluation in traditional research. The UGS recreational situation extracted from the mobile phone signaling data and accessibility evaluation results was used to calculate the UGS supply and demand deviation to further elucidate the fairness of UGS spatial distribution and optimization strategies.

## Materials and methods

### Research area

Hefei, located in east China, is a sub-center city of urban agglomeration and a national science center, with a total area of 11,445 km^2^ and a population of 9.36 million (China National Bureau of Statistics, 2020). The government of Hefei has made considerable efforts in ecological construction. After the 1956 edition of the urban master plan, a ring-city park system and a fan-shaped urban structure were formed. Hefei was selected as one of the first national garden cities of China in 1992. By the end of 2020, Hefei city's forest coverage rate reached 28.36% of the total area, with a forest volume of 10.6 million m^3^, while the urban built-up area green coverage rate reached 46%. The green area per capita was 13 m^2^, far more than the minimum UGS requirement of 9 m^2^ per capita recommended by the World Health Organization. Although the greening level of Hefei is higher than that of other surrounding cities, more in-depth UGS accessibility evaluation research is needed for the future optimization of UGS.

**Figure 1:**
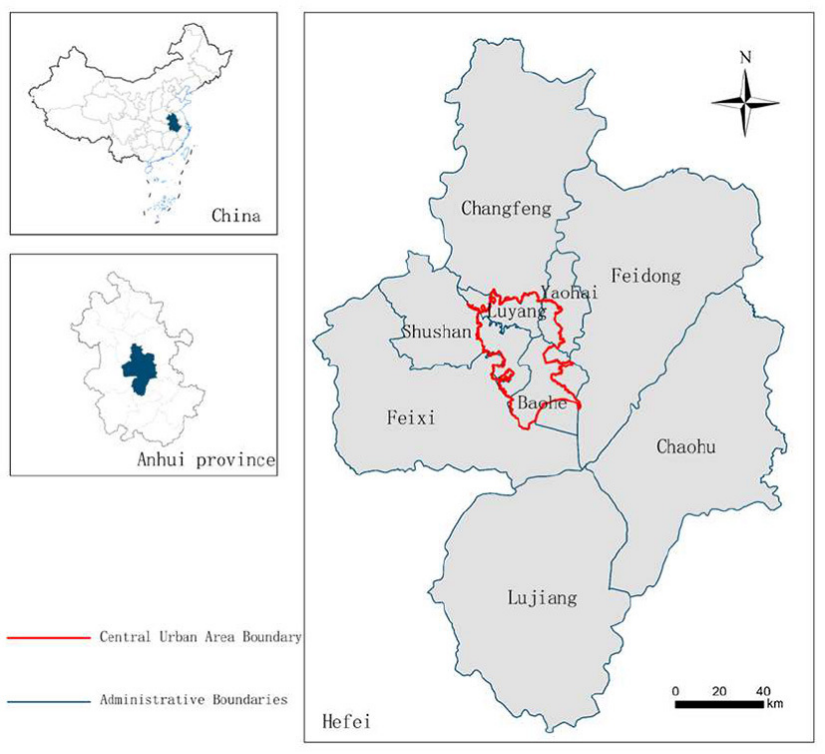
Central urban area of Hefei.

**Figure 2:**
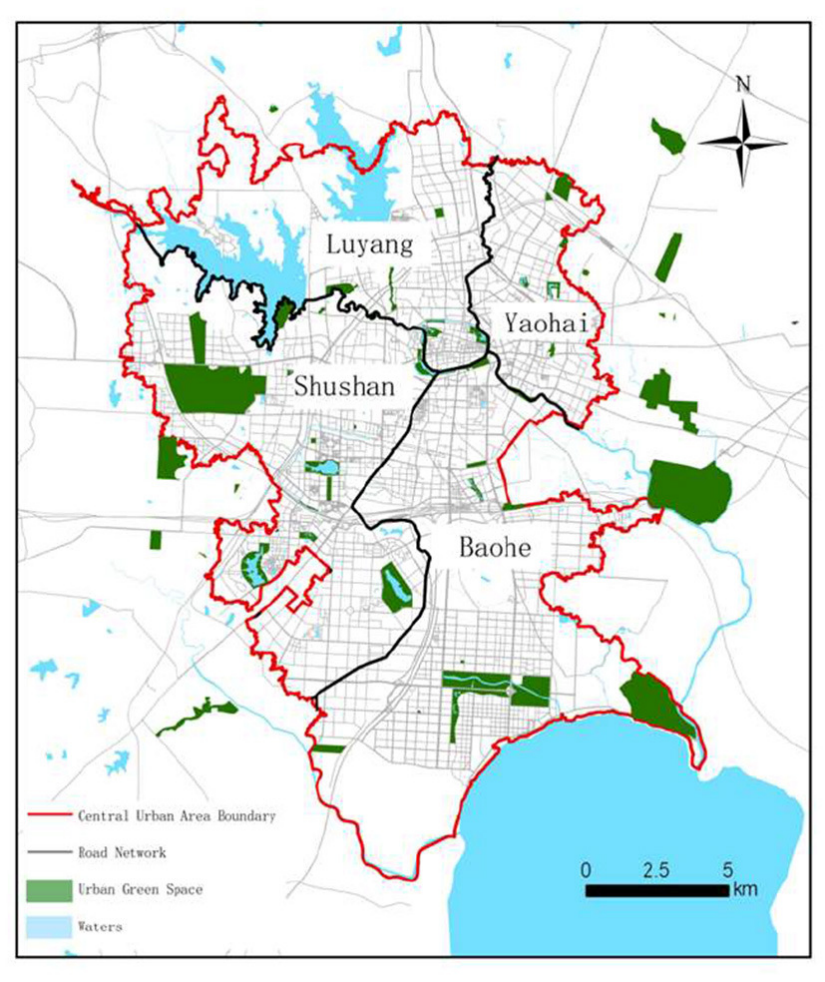
Distribution of UGS in central urban area of Hefei.

This study takes the central urban area of Hefei as the research area (Figure 1) and the public parks in the central urban area of Hefei as the UGS research objects (Figure 2). The central urban area of Hefei has a total area of about 486 km^2^, according to the definition of the central urban area of Hefei City Master Plan (2011–2020).

### Data source and processing

#### UGS data

The UGS data used in this paper were derived from the Gaode Map. Using the Hefei Green Space System Planning (2007–2020) and Hefei City Master Plan (2011–2020), combined with the map and field survey, the spatial location, area, and quantity of the UGS in the central urban area and its surrounding area within a distance of 15,000 m were further verified. A total of 75 parks were obtained and considered to be UGS (Table 2).

**Table 2:** Summary of UGS in the central urban area of Hefei.

**District**	**Comprehensive park /hm^2^**	**Theme park /hm^2^**	**Community park /hm^2^**	**Garden /hm^2^**	**Total area of the park /hm^2^**
Luyang	92.39	37.89	58.73	5.33	194.34
Shushan	1,106.54	263.23	65.69	18.77	1,454.23
Yaohai	69.98	12.19	34.34	5.06	121.57
Baohe	966.57	45.64	57.7	3.01	1,072.92
Total	2,235.48	358.95	216.46	32.17	2,843.06
Proportion	(78%)	(13%)	(8%)	(1%)	(100%)

We converted UGS surface elements to point elements in ArcGIS 10.2. The centroid of the park with an area of <100 hm^2^ was taken as the supply point, and the coordinates of the entrances and exits of UGS equal to or >100 hm^2^ were considered as supply points for the accessibility evaluation (35). To avoid the repeated calculation of the area, UGS equal to or >100 hm^2^ were also decomposed accordingly when the entrance and exit coordinates were taken as its supply points, which can reduce the error of accessibility calculation. Finally, 80 supply points of the UGS were obtained.

#### Residential population and visitor data

The precision of the population statistics has a direct impact on the evaluation results of UGS accessibility (36). It is difficult to use traditional statistical data, such as census data, to analyze the behavioral patterns of different populations at a fine scale due to a large scale of statistical units (37). Compared with traditional statistical data, mobile phone signaling data have a higher accuracy and can reflect massive and real-time population activities, and these data have been widely used in urban population activity research, such as work-housing commuting behavior, urban dynamic space identification, and traffic efficiency evaluation (38). We used the mobile signaling data of China Unicom users in May 2021 to identify the residents in the central urban area by screening the users who have stayed in Hefei for over 10 days. Finally, we identified 929,916 residents from the China Unicom mobile phone signaling database, accounting for 18.17% of the total population of central urban residents.

When the user's mobile phone communicates with the mobile phone signaling base station of China Unicom, the base station will locate the user and record the user interaction information, including user ID, interaction time, base station code, and territory code (Table 3). There are about 13,000 China Unicom mobile phone signaling base stations in Hefei's central urban area, with distance intervals of 250–1,000 m.

**Table 3:** Sample data of interaction information collected by mobile phone signaling base station.

**User ID**	**Interaction time 1 (the beginning of an event)**	**Interaction time 2 (the end of an event)**	**Base station code**	**Territory code**
773068*****3505730	2021-5-1 0:15	2021-5-1 9:56	7007403516115268	340104
777258*****8329382	2021-5-1 7:56	2021-5-1 18:38	70071403728115696	340104
772948*****5537730	2021-5-1 11:42	2021-5-1 15:29	7007403420115260	340104
…	…	…	…	…

**Figure 3:**
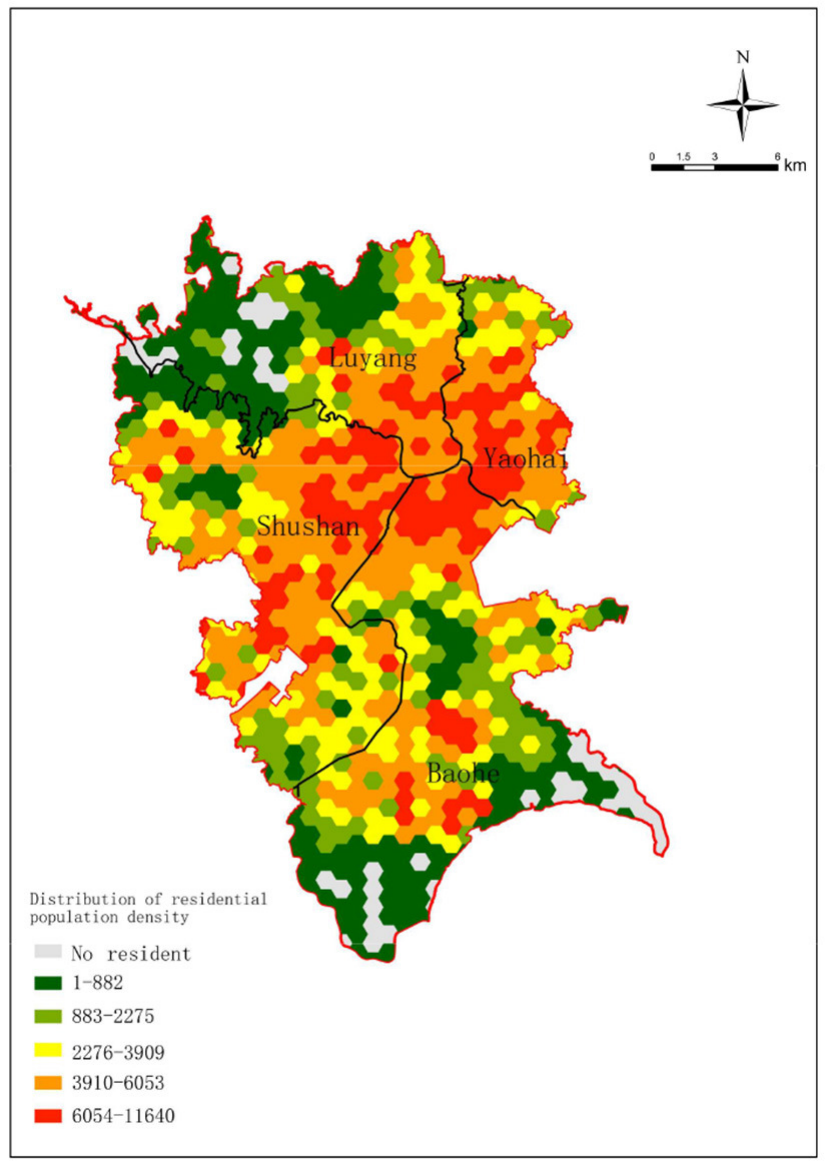
Distribution of the residential population density in the central urban area of Hefei.

In relevant studies, a regular hexagon, which has the same distance from point to centroid in six directions, can reduce the sample deviation caused by the boundary effect of grid shape and is suitable for spatial analysis (39, 40). Therefore, we used a regular hexagon grid with a lateral length of 500 m to establish the honeycomb grid network in ArcGIS10.2 and aggregated the areas falling within the regular hexagon into the centroid of the grid. On this basis, the spatial analysis honeycomb network was formed, and 827 hexagon grids were obtained. We assigned the corresponding attribute information to the grid according to the land use category in the grid and identified the residential grids and the UGS grids. The identification standard of the residential grid was that there is a residential population within the grid range, and the identification standard of the UGS grid was that the centroid of the grid falls in the UGS area. The same grid can be identified as both a residential grid and a UGS grid at the same time. While the UGS grids are used for UGS recreational visitor identification, the centroids of the residential grids are used as the demand points in the accessibility evaluation. The residential grids are used as the basic units for both accessibility evaluation and supply-demand deviation evaluation. By collecting data on the places where the residents were at from 21:00 P.M. to 08:00 A.M. the next day and marking the places with the longest stay as the places of residence, the population of each place was included in the residential grid that each of the places was in. The distribution map of the residential population density in the central urban area of Hefei is shown in Figure 3.

To acquire the deviation between the theoretical accessibility and actual recreational activities in the UGS, we first needed to identify UGS visitors who visited UGSs in the month and were in the UGS grids for 30 min or more from 8:00 A.M. to 21:00 P.M. Then, the number of residents who have been to a UGS once or more than once in each grid was counted for the supply and demand deviation calculation.

#### Travel mode and travel distance data

If the speed of UGS visitors was 0–5 km/h, then they were considered to be in walking mode. Visitors with speeds of 5–15 km/h and 15–60 km/h were considered to be in riding mode and driving mode, respectively. There were 244,017 residents who went to UGS in May 2021 and made 801,311 trips to UGS, including 72,622 walking trips, 147,530 cycling trips, and 581,159 driving trips, accounting for 9.06, 18.41, and 72.53% of the total trips, respectively. The proportion data of the different travel modes to UGS were used as the weight basis for the accessibility evaluation.

The shortest travel path from each demand point to the supply point of the UGS was obtained through the path planning interface in the Gaode API (application programming interface). First, by using the coordinate conversion interface, the GPS coordinates of the supply point and the demand point were transformed into the Goethe coordinates. Then, by using the path planning interface and taking the coordinates of the origin and destination (OD) points as parameters, the path planning requests of walking, cycling, and driving were initiated to obtain the distance data under different travel modes as the actual distance from each supply point to the demand point (Table 4).

**Table 4:** Sample data of the travel distance between UGS and residential areas.

**UGS number**	**Residential grid number**	**Walking distance**	**Cycling distance**	**Driving distance**
001	001	2,653 m	3,055 m	3,070 m
001	002	8,662 m	9,633 m	9,656 m
001	003	7,552 m	8,873 m	8,900 m
……	……	……	……	……

### Methodology

#### Implementation of Ga2SFCA

In this study, the commonly used 2SFCA method is improved in terms of the data source and OD cost calculation rules. The Gaussian function decays slowly when approaching the search threshold, which is more in line with the actual travel situation of residents (41). Therefore, we used the Gaussian two-step floating catchment area (Ga2SFCA) method to calculate UGS accessibility, which includes the following steps:

Step 1: For each supply point *j* of the UGS, all residents' demand points *k* with *j* as the center and *d*_0_ as the threshold range are searched. For the population of these demand points, the Gaussian function is used for the attenuation calculation, and then, the sum is obtained. Finally, the area of the supply point is divided by the sum of the population of the demand point to obtain the supply-demand ratio *R*_*j*_ of the UGS *j*, which is the service capacity value of the supply point of the UGS. The calculation formula is as follows:

(1) $$\begin{array}{l} R_{j}=\frac{S_{j}}{\sum_{k\in \left\{d_{kj}\leq d_{0}\right\}}G\left(d_{kj},d_{0}\right)\times P_{k}} \end{array}$$

where *S*_*j*_ represents the supply scale of UGS *j*, measured by its area; *d*_*kj*_ represents the travel time from residential area *k* to park green space *j*; and *P*_*k*_ is the scale of demand for residential area *k* in the search range, measured by population.

*G* (*d*_*kj*_, *d*_0_) is a Gaussian function, and the calculation formula is as follows:

(2) $$\begin{array}{l} G\;\left(d_{kj},d_{0}\right)=\{\begin{array}{cc} e^{{-\frac{1}{2}\times \left(\frac{d_{kj}}{d_{0}}\right)}^{2}}-e^{-\frac{1}{2}},d_{kj}\leq d_{0}\\ 0^{1-e^{-\frac{1}{2}}},\;\\ \; & d_{kj}>d_{0} \end{array} \end{array}$$

Step 2: For each residential grid *i*, all UGS *j* values with *i* as the center and *d*_0_ as the threshold range are searched. Similarly, the supply-demand ratio *R*_*j*_ of each UGS is calculated and summed by Gaussian function attenuation. Finally, the accessibility index *A*_*i*_ of each residential area *i* is obtained. The calculation formula is as follows:

(3) $$\begin{array}{l} A_{i}=\underset{i\in \left\{d_{ij}\leq d_{0}\right\}}{\sum }G\left(d_{ij},d_{0}\right)\times R_{j} \end{array}$$

where *R*_*j*_ is the supply and demand ratio of park green space j in the threshold range; and *d*_*ij*_ is the walking time between residential area *i* and UGS *j*. *A*_*i*_ represents the accessibility index of UGS of residential area *i*, and the larger the value, the higher the accessibility of UGS.

Step 3: The accessibility index of UGS under different traffic modes is calculated for each residential area *i*. The accessibility index is overlayed according to the proportion of various traffic modes used by permanent residents in the central urban area in May 2021 to obtain the comprehensive accessibility *C*_*i*_ of each residential area *i*. The larger the calculated *C*_*i*_, the better the comprehensive accessibility of residential area *i*. The calculation formula is as follows:

(4) $$\begin{array}{l} C_{i}=\underset{n\in \left\{1,2,3\right\}}{\sum }W_{n}\times A_{in} \end{array}$$

where *W*_*n*_ is the proportion of traffic mode *n* used by permanent residents in the central urban area in May 2021, and *A*_*in*_ represents the accessibility index of UGS in the traffic mode *n* of residential area *i*.

Considering that residents have different traffic modes to go to the UGS and are willing to bear different travel time costs, we divided the search thresholds into six categories, namely, the walking comfort threshold, cycling comfort threshold, driving comfort threshold, walking limit threshold, cycling limit threshold, and driving limit threshold. Combined with the average speed of various traffic modes, the final search threshold was determined, as shown in Table 5.

**Table 5:** Summary of accessibility search thresholds for UGS.

** 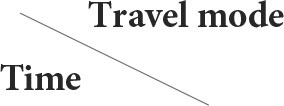 **	**Walking**	**Cycling**	**Driving**
15 min	1,250 m	3,750 m	7,500 m
30 min	2,500 m	7,500 m	15,000 m

#### Overlay analysis of accessibility and UGS recreational characteristics

We obtained the UGS visitor data from the mobile phone signaling data and analyzed the coupling matching degree between the number of UGS visitors and the accessibility level results to explore the actual situation of the UGS supply and demand relationship. We used the normalized value of the number of residents who have visited UGS between May 9^th^ and 15^th^ and lived in residence *i* as the demand for UGS and the accessibility level as the supply of UGS. Then, the two were overlayed to calculate the supply and demand ratio. The spatial distribution of the measured results was visualized to visually examine the spatial difference between the UGS supply and demand in the central urban area of Hefei. The calculation formula is as follows:

(5) $$\begin{array}{l} Z_{i}=\frac{A_{i}/A}{P_{i}/P} \end{array}$$

where A represents the sum of the accessibility levels of all grids in the central urban area, *P*_*i*_ is the number of visitors who have visited UGS between May 9^th^ and 15^th^ and lived in residential area *i*, and *P* denotes the total population of all UGS visitors in the central urban area. *Z*_*i*_ represents the deviation between the supply and demand of UGS. The calculation results between 0 and 0.8 were considered as “low supply–high demand” areas; 0.8–1.2 were considered “supply–demand balance” areas; and above 1.2 were considered “high supply–low demand” areas.

## Results

### Accessibility analysis

**Figure 4:**
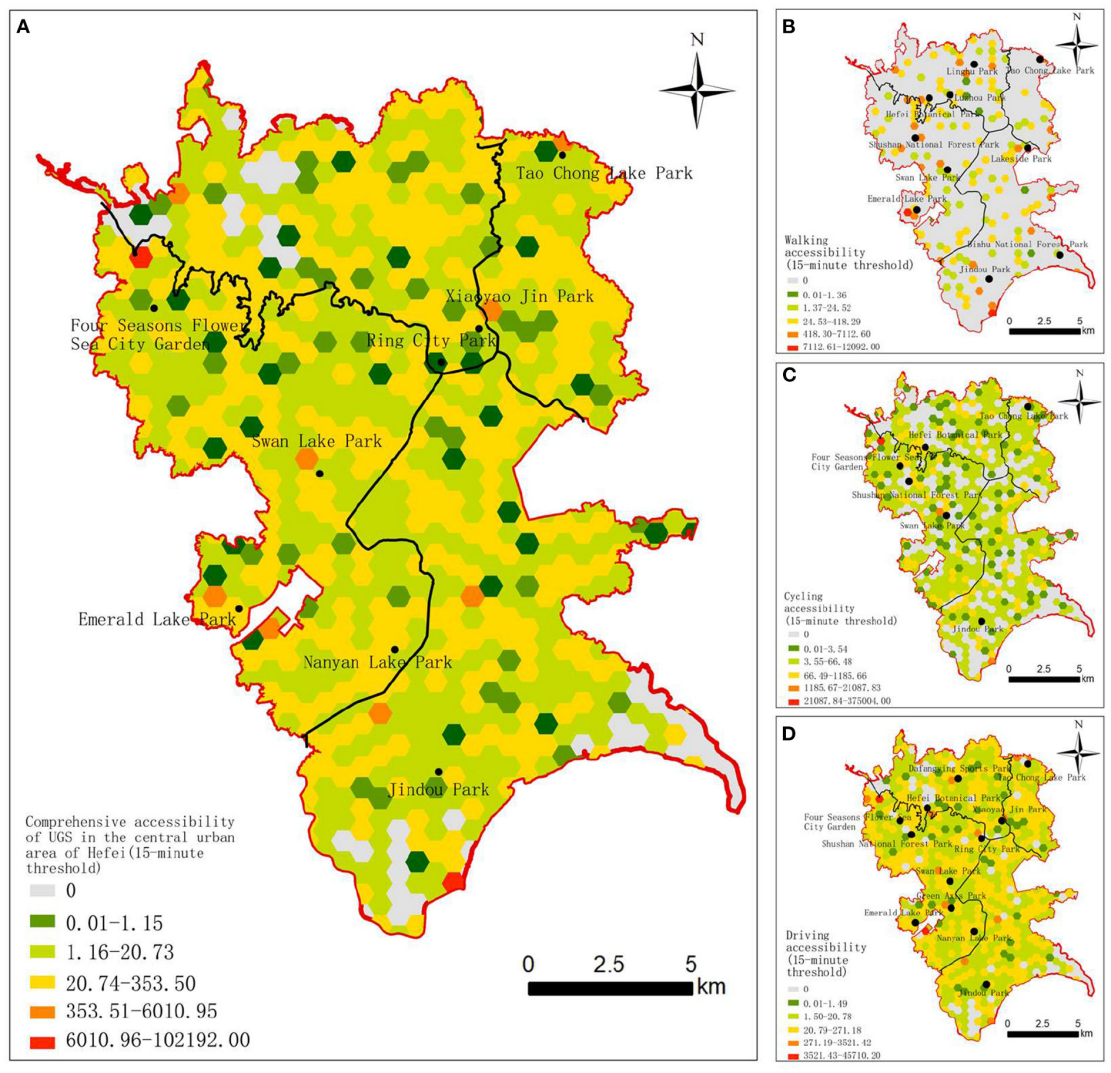
Accessibility of UGS in the central urban area of Hefei at the 15-min threshold. **(A)** Comprehensive accessibility. **(B)** Walking accessibility. **(C)** Cycling accessibility. **(D)** Driving accessibility.

**Figure 5:**
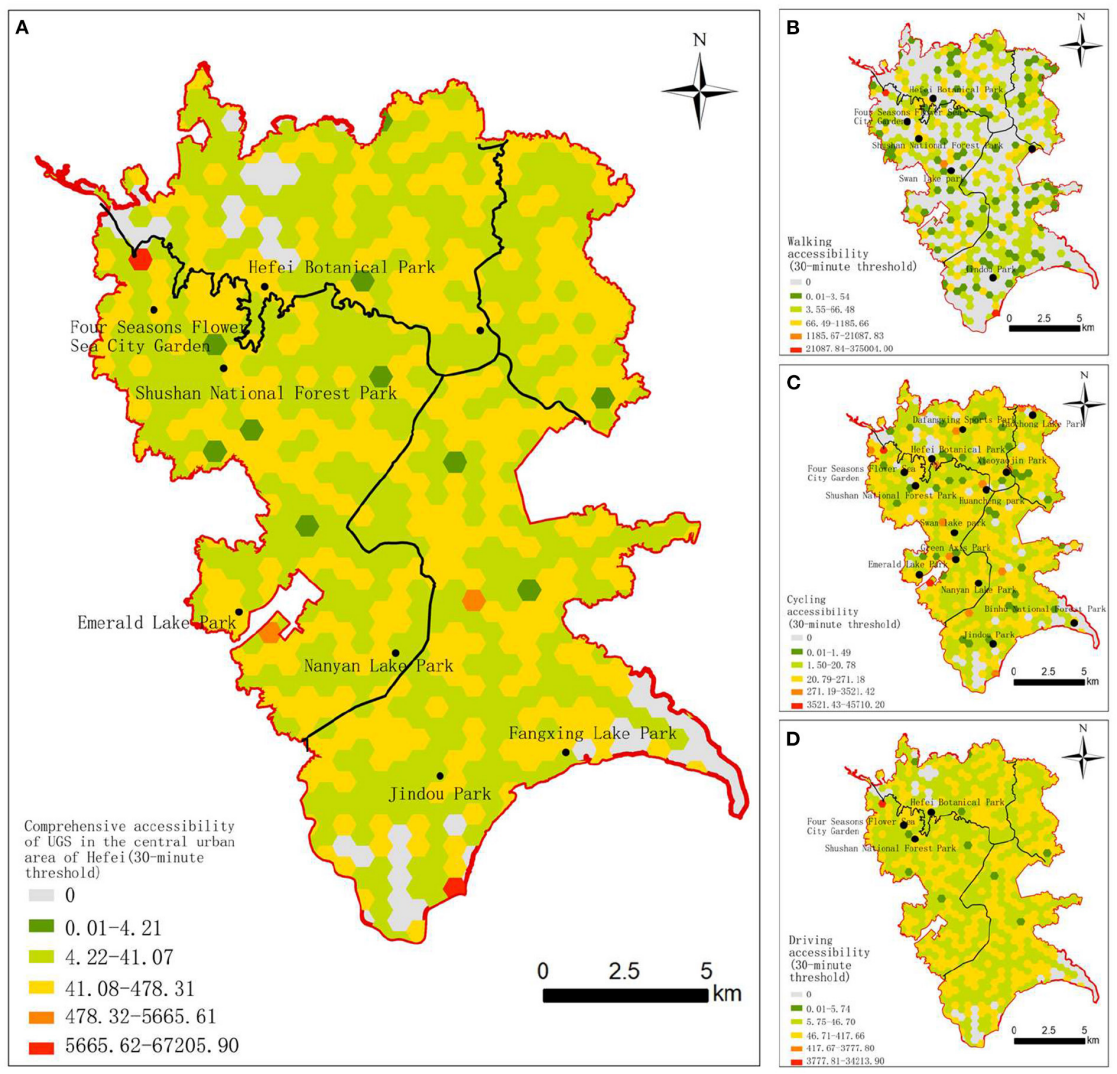
Accessibility of UGS in the central urban area of Hefei at the 30-min threshold. **(A)** Comprehensive accessibility. **(B)** Walking accessibility. **(C)** Cycling accessibility. **(D)** Driving accessibility.

In this study we used the Ga2SFCA model to calculate the accessibility of the three travel modes under different time thresholds; then, the accessibility results of three travel modes were overlayed according to the weight values obtained from the proportion data of the different travel modes. Based on the results of the above analysis, the comprehensive comfort accessibility and comprehensive limit accessibility were obtained. The standardized results were visualized in ArcGIS10.2 and divided into five grades according to the geometric interval, representing the lowest, low, moderate, high, and highest accessibility scores (Figures 4, 5).

Figures 4, 5 shows that there is an obvious uneven distribution of UGS resources in the central urban area of Hefei. In general, with the increase in the time threshold or travel distance, the number of grids above the moderate accessibility level generally increases, and the spatial distribution of the grids with moderate, high and highest accessibility level present different patterns of contiguous, clusters, and spots distribution, respectively. Specifically, the accessibility of the surrounding areas of the Four Seasons Flower Sea City Garden, the Emerald Lake Park, the Nanyan Lake Park, and the Jindou Park at different time thresholds and different travel modes is much higher. The accessibility of UGSs in the western Yaohai District, the southeastern Luyang District, the northeastern Shushan District, and the northern Baohe District is poor, mainly because these areas are old urban areas with a relatively dense population and a large demand for UGS.

The accessibility of UGS under different thresholds was analyzed based on the residential grid scale. (1) At the 15-min threshold, the areas with a moderate or higher walking comfort accessibility level are small, accounting for about 11.85% of all the grids, and are mainly distributed in spot patterns along the periphery of the UGSs. The areas with a moderate or higher cycling comfort accessibility level are slightly more than those with walking comfort accessibility, accounting for about 13.06% of all the grid units, and show a cluster distribution pattern. The areas with a moderate or higher driving comfort accessibility level are far larger than those with walking and cycling comfort accessibility, accounting for about 48.25% of all the grid units, among which the moderate-level areas are regularly distributed all around the central urban area, and the high-level areas are mainly distributed in the west of the central urban area, showing a contiguous distribution pattern along the Four Seasons Flower Sea City Garden, the Shushan National Forest Park, the Swan Lake Park, the Ring City Park, the Green Axis Park, the Emerald Lake Park, and the Nanyan Lake Park. (2) At the 30-min threshold, there are relatively fewer areas with a moderate or higher level of walking comfort accessibility, accounting for about 11.97% of all the grids, mainly distributed in linear patterns along the periphery of the UGSs. The areas with moderate or higher cycling accessibility were far greater than the former, accounting for about 48.25% all the grid units, mainly distributed regularly around the west part of the central urban area. The areas with a moderate or higher level of driving accessibility are fewer than those with a moderate or higher level of cycling accessibility, accounting for about 45.22% of all the grid units, and the moderate-level areas are distributed regularly, while the high-level areas have fewer numbers and mainly distributed in the northwest part of the central urban area. The high level of comprehensive comfort accessibility with the 15-min threshold is distributed around the Four Seasons Flower Sea City Garden, the Xiaoyao Jin Park, the Swan Lake Park, the Taochong Lake Park, the Emerald Lake Park, the Jindou Park, and the Nanyan Lake Park, accounting for about 1.45% of all the grid units. The Four Seasons Flower Sea City Garden is located in the western suburbs of Hefei, about 10 km from the center of the old urban area. The road network around the Four Seasons Flower Sea City Garden is relatively developed, and the living density of the surrounding population is moderate; so, the comprehensive accessibility is high. The Jindou Park, the Emerald Lake Park, and the Tao Chong Lake Park are surrounded by other UGSs, with a lower residential population. Although the Xiaoyao Jin Park and the Swan Lake Park are surrounded by high-density residential population, their better green space construction and road system planning make up for the accessibility level. The low comprehensive comfort accessibility areas are distributed in the west part of the Yaohai District, the south part of the Luyang District, the northeast part of the Baohe District, and the north of the Shushan District. There are few UGSs but a large number of residential areas in the above regions, and the road network connectivity is poor, all leading to low comprehensive comfort accessibility.

The high and highest comprehensive limit accessibility levels with the 30-min threshold are distributed around the Four Seasons Flower Sea City Garden, the Emerald Lake Park, the Nanyan Lake Park, and the Jindou Park, accounting for about 0.48% all the grid units, which is slightly less than the comprehensive comfort accessibility with the 15-min threshold. The areas with a low comprehensive limit accessibility are distributed in the west part of the Yaohai District, the southeast part of the Luyang District, the northeast part of the Shushan District, and the north part of the Baohe District. With the increase in the general travel distance of users in the central urban area, the comprehensive limit accessibility shows a significant regular distributed phenomenon, and the number of areas with the highest and lowest grades decreased significantly.

### Analysis of supply and demand deviation

**Figure 6:**
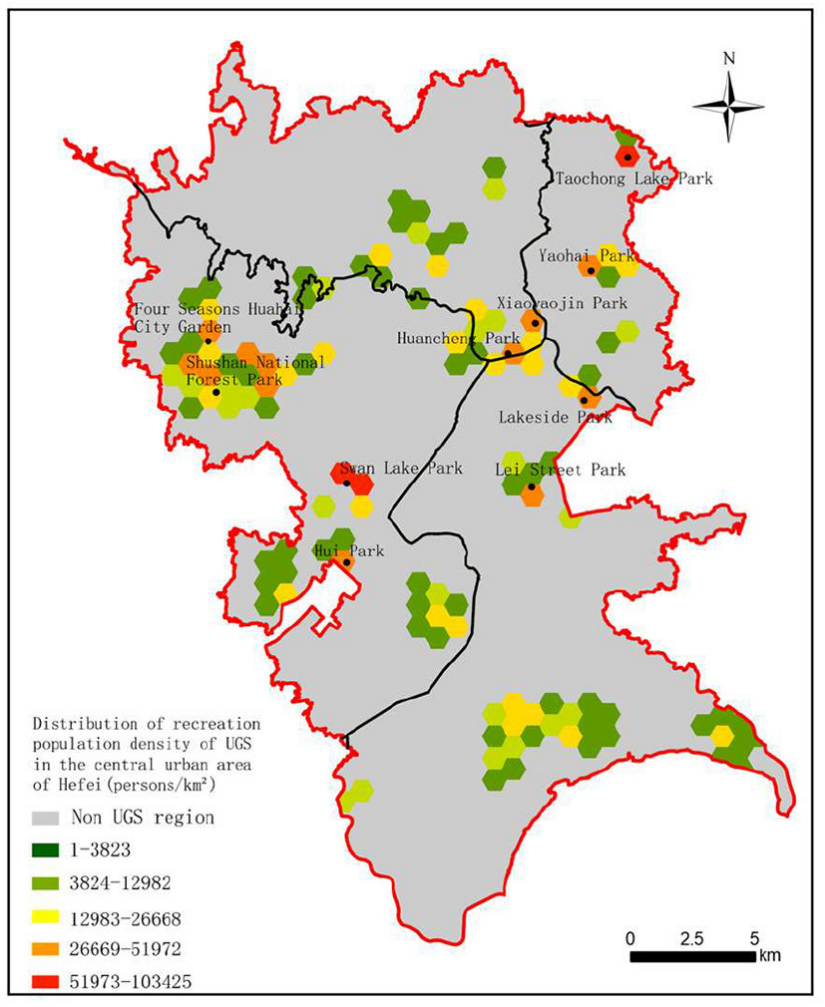
Distribution of the UGS recreational population density in the central urban area of Hefei.

According to the distribution of the UGS recreational population density in the central urban area of Hefei (Figure 6), it can be concluded that there are 10 parks favored by the central urban population for UGS recreational activities, namely the Swan Lake Park, the Taochong Lake Park, the Shushan National Forest Park, the Four Seasons Flower Sea City Garden, the Ring City Park, the Xiaoyao Jin Park, the Yaohai Park, the Binhu Lakeshore National Park, the Leijie Park, and the Huiyuan Garden.

**Figure 7:**
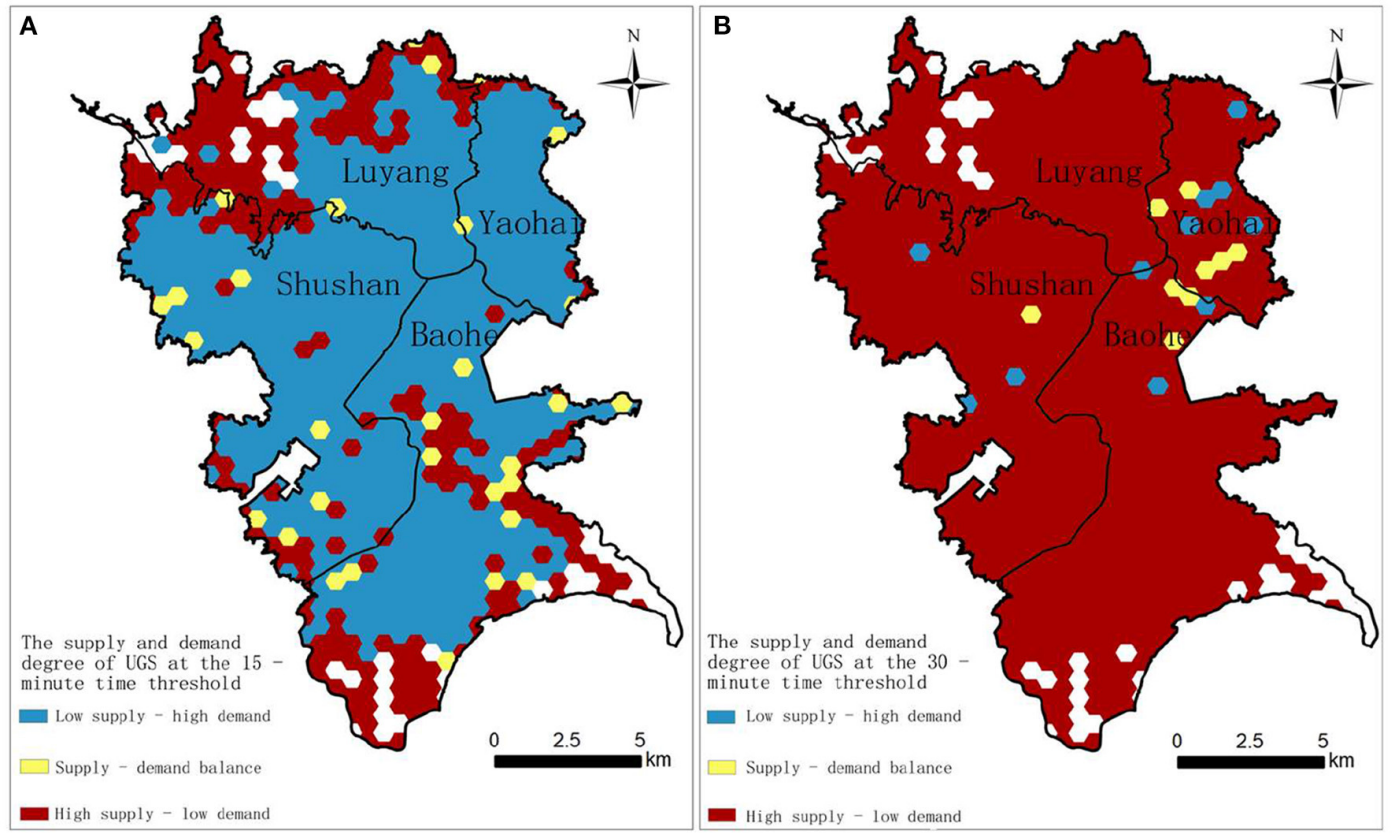
Deviation between supply and demand of UGS at different time thresholds. **(A)** At the 15-min time threshold; **(B)** At the 30-min time threshold.

Using Formula 5, the hexagonal grid was used as the statistical unit to calculate the deviation between supply and demand of UGS at different time thresholds (Figure 7). In the grid, the China Unicom users without UGS recreational demand in May 2021 were not included in the consideration of the deviation of supply and demand of UGS, and this result appears as the blank area.

According to results, for the 15-min threshold, the overall demand of the central urban area is greater than the supply, and the proportion of grid units where demand exceeds supply accounts for about 63.97% of all the grid units. The number of grids with a balanced supply and demand of UGS is small and scattered, accounting for about 3.75% of all the grid units. The grid units where supply exceeds demand account for about 32.29%, and these are mainly distributed in the north and south part on the edge of the central city near the suburbs. For the 30-min threshold, the central urban area shows a pattern of supply being greater than demand, but the proportion of areas with balanced supply and demand decreased, accounting for 1.09% of all the grid units.

Further analysis was carried out based on the distribution of the recreational population density of UGS in the central urban area of Hefei. First, the old urban area is the center of the city, which has a high residential population density and low per capita green space, which are the main reasons for the deviation of the supply and demand of the UGS. Second, compared with the old urban area, the northern Shushan District and southern Baohe District are both newly built and have better UGS construction, better road infrastructure, and low population density, which leads to a lower demand than the old urban area, resulting in the supply exceeding demand. Third, there are many comprehensive parks near the periphery of the old urban area, and residents are generally more inclined to use these comprehensive green spaces. The abovementioned comprehensive effects lead to the contradiction between the supply and demand of accessibility of UGS in the urban area.

## Discussion

In this study, mobile phone signaling data were used instead of traditional statistical data to obtain accurate population positioning data. The distance between supply and demand points was represented by the actual distance of the Gaode Map path planning tool, which strengthened the data granularity and improved the accuracy of the evaluation results. The optimized Ga2SFCA method was used to measure the accessibility level under different transportation modes, and the actual traffic mode proportion data were used to calculate the comprehensive accessibility level. In addition, two different search distances for the time threshold were set based on residents' comfort and limited travel time. The accessibility differences under different travel modes and time thresholds were discussed. Finally, we combined the results of the UGS accessibility evaluation with the actual use of UGS to analyze the difference between supply and demand. In this study, we focused on the application of mobile phone signaling data in measuring the actual usage of UGS. Based on mobile signaling data, we acquired the precise resident positioning data in the residential area and the actual recreational activities in the UGS. Combined with the path planning interface in the Gaode API, with the support of refined travel data, we can analyze the coupling matching degree between the number of UGS visitors and the accessibility level results to explore the actual situation of the UGS supply and demand relationship. The use of fine-grained data and the improvement of the 2FSCA method are the most important contributions of this study.

In general, the grids with high accessibility to the UGSs in the central urban area of Hefei tend to be distributed near large urban parks, and this phenomenon is more obvious in the evaluation results of walking and cycling accessibility at the 15-min time threshold. However, with the increase in the time threshold and travel distance, the accessibility evaluation results gradually show a regular distributed pattern. In addition, the accessibility level of the driving mode at the 15-min time threshold and the walking and cycling mode at the 30-min time threshold shows that the number of grids above the moderate level increases significantly. For some socially vulnerable groups, especially low-income groups and the elderly, the ownership of a private car and the ability to undertake long-distance travel are clearly unfair conditions for access to more UGSs (42, 43). Therefore, it is still necessary to increase the area of UGSs to increase the opportunities for urban residents to obtain green space resources and ensure fairness of access to UGS for socially vulnerable groups. When urban land resources are limited and incremental expansion of old urban areas is difficult, decision-making departments should first consider improving the quality of existing UGSs. In densely populated areas with limited vacant land, smaller street gardens, pocket parks, and rooftop gardens should be considered to improve the accessibility of the UGS. Decision-makers should increase the number of large park entrances and strengthen the construction of roads around comprehensive parks.

In addition, China's government recommends that residents stick to low-carbon travel modes, which means giving priority to walking, cycling, and public transportation. For the old urban area, where there is limited urban construction land, it is undoubtedly a better choice to further develop the low-carbon transportation system than to increase the green area of the park. The construction of pedestrian and cycling networks, as well as the public transportation system, should be strengthened to improve the connectivity between residential areas and UGSs. According to the specific situation, decision-making departments should consider increasing the residential land area around UGSs to improve the utilization rate of UGSs in the new urban area, where the resources are sufficient.

## Conclusion

The fairness of the UGS layout is the basic guarantee of urban residents' quality of life. Based on the optimized Ga2SFCA method, we evaluated the spatial accessibility of the existing UGS in the central urban area of Hefei under different travel modes and analyzed the supply and demand deviation of the UGS combined with the actual recreational population. The results show the following:

(1) Setting different travel modes and different time thresholds significantly affects the evaluation results of the UGS accessibility level. At the 15-min threshold, the areas with a moderate or higher walking comfort accessibility level are small, and the areas with a moderate or higher driving comfort accessibility level are far larger than those with walking and cycling comfort accessibility. At the 30-min threshold, there are relatively fewer areas with a moderate or higher level of walking comfort accessibility, while the areas with moderate or higher cycling accessibility were far greater than the former. The spatial distribution of UGSs in the central urban area of Hefei is uneven. In general, with the increase in the time threshold, the number of grids above the moderate accessibility level generally increases, and the spatial distribution of the grids with moderate, high and highest accessibility level present different patterns of contiguous, clusters, and spots distribution respectively. The accessibility of UGSs in old urban areas is poor, mainly because these areas have a relatively dense population and a large demand for UGS. Due to the shortage of construction land, it is difficult to add new UGS, even if the problem of the relatively small number of UGSs becomes more severe.

(2) The characteristics of supply-demand deviation of UGSs are revealed by analyzing the coupling matching degree between the number of UGS visitors and the accessibility level results. At the 15-min threshold, the demand of UGSs in the central urban area is greater than the supply, but at the 30-min threshold, the supply exceeds the demand. In addition, at the two different time thresholds, the number of grids with balanced supply and demand is small and the grids are scattered, mostly distributed around large comprehensive parks. Such areas usually have moderate residential density and good road connectivity. The grids with balanced supply and demand or more UGS supply than demand are mainly concentrated in the southern area and the edge of the central city near the suburbs. All these areas have large, comprehensive parks. The population density of the surrounding communities is moderate, and the road connectivity is better, with a higher level of transportation infrastructure than that in the old urban area. The southern area is the new urban area established by Hefei City, which has more capital and land for the construction of UGSs and various urban infrastructure, and the construction level of living environment is relatively high. At the same time, an increasing number of people choose to live in the new urban area, which further brings capital and population vitality and reduces the supply-demand deviation. Due to the increasing urban population, the population density of the old urban area is not decreased, which makes UGS resources scarce. The government should pay attention to improving the existing quality of UGSs, as well as alleviating UGS inequality by using small vacant lands and inefficiently developed lands to increase the area of UGSs.

Refined and scientific evaluation of UGS accessibility is an important foundation for the accurate restoration of urban green space fairness. Based on the refined mobile phone signaling data, we can obtain the precise accessibility evaluation results and actual supply and demand situation, which can certainly help the government to understand the status of the actual park usage. For the area with imbalanced supply and demand, the government should carry out thematic studies and further explore the reasons. This study can provide guidance for the government to formulate fair green space policies, advocate to guarantee access to green space resources for the socially vulnerable groups, and finally promote the healthy and sustainable development of cities. This study also had some limitations, largely due to the availability of data. When calculating the supply capacity of UGS, we did not consider the internal facilities, service quality, and service facilities of UGS. In the calculation of UGS demand, only the population number of the statistical unit was considered, without an in-depth consideration of the needs of people with different social attributes, which can be significant (44). A discussion on the causes of the discrepancy between the supply and demand of accessibility is needed and will be further explored in future research.

## Data availability statement

The data analyzed in this study is subject to the following licenses/restrictions: The urban green space data were derived from the Gaode API (application programming interface) (https://lbs.amap.com/), which is publicly available. The travel mode and travel distance data was also obtained through the path planning interface in the Gaode API. Additionally, the residential population and recreational visitor data were extracted from the China Unicom mobile phone signaling database (http://daas.smartsteps.com), which is not publicly available, and, after logging into the database system, the data can be retrieved and statistically analyzed by using structured query language. Requests to access these datasets should be directed to Gaode API (application programming interface) (https://lbs.amap.com/); China Unicom mobile phone signaling database (http://daas.smartsteps.com).

## Author contributions

JC and CW contributed to the conception and design of the study. CW performed the statistical analysis and wrote the first draft of the manuscript. JC wrote sections of the manuscript. YZ and DL contributed to funding acquisition. All authors contributed to manuscript revision, read, and approved the submitted version.

## Funding

This research received financial support from Anhui Agricultural University (Grant No. RC372016 and No. K2137001) and Anhui Provincial Department of Science and Technology (Grant No. 202004a06020014).

## Conflict of interest

The authors declare that the research was conducted in the absence of any commercial or financial relationships that could be construed as a potential conflict of interest.

## Publisher's note

All claims expressed in this article are solely those of the authors and do not necessarily represent those of their affiliated organizations, or those of the publisher, the editors and the reviewers. Any product that may be evaluated in this article, or claim that may be made by its manufacturer, is not guaranteed or endorsed by the publisher.
